# MicroRNAs and Gene Regulatory Networks Related to Cleft Lip and Palate

**DOI:** 10.3390/ijms24043552

**Published:** 2023-02-10

**Authors:** Chihiro Iwaya, Akiko Suzuki, Junichi Iwata

**Affiliations:** 1 Department of Diagnostic & Biomedical Sciences, School of Dentistry, The University of Texas Health Science Center at Houston, Houston, TX 77054, USA; 2 Center for Craniofacial Research, The University of Texas Health Science Center at Houston, Houston, TX 77054, USA; 3 The University of Texas MD Anderson Cancer Center UTHealth Houston Graduate School of Biomedical Sciences, Houston, TX 77030, USA

**Keywords:** cleft lip, cleft palate, microRNA, craniofacial development, environmental factor

## Abstract

Cleft lip and palate is one of the most common congenital birth defects and has a complex etiology. Either genetic or environmental factors, or both, are involved at various degrees, and the type and severity of clefts vary. One of the longstanding questions is how environmental factors lead to craniofacial developmental anomalies. Recent studies highlight non-coding RNAs as potential epigenetic regulators in cleft lip and palate. In this review, we will discuss microRNAs, a type of small non-coding RNAs that can simultaneously regulate expression of many downstream target genes, as a causative mechanism of cleft lip and palate in humans and mice.

## 1. Introduction

Congenital anomalies are a major cause of infant and childhood morbidity, affecting 2–3% of all babies. Cleft lip with/without cleft palate (CL/P) is one of the most prevalent congenital birth defects; it affects 1 in 500 babies in Asian and Native American populations, 1 in 1000 in European-derived populations, and 1 in 2500 in African-derived populations [1]. In the US, cleft lip only (CLO) occurs in 1 in 2800 babies, cleft palate only (CPO) in 1 in 1700 babies, and CL/P in 1 in 1600 babies. A total of 30% of cases of CL/P are syndromic; its etiology is complex with multifactorial effects. For non-syndromic CL/P, it is estimated that 30–50% of cases are caused by genetic factors, and 50–70% are due to non-genetic factors such as abnormal maternal conditions and exposure to teratogens [2,3,4,5]. Individuals with CL/P require multidisciplinary, long-term care from birth to adulthood, with an estimated lifetime cost of more than USD 150,000. Thus, these individuals are affected not only aesthetically and functionally (e.g., at the level of pronunciation, swallowing and suckling), but also economically.

Mice have been frequently used to study craniofacial morphogenesis and its underlying cellular and molecular mechanisms because their developmental processes are similar to those of humans and occur within a short window of time. Given these advantages, genetic mutant mouse models and in vivo cell lineage-tracing methodologies have been used to identify cellular and molecular mechanisms related to CL/P. Upper lip formation begins with enlargement of the maxillary processes (MxPs), which develop from the first pharyngeal arch at the lateral boundary of the stomodeum at embryonic day 9.5 (E9.5) in mice and gestation day 28 in humans [6]. At E10.0 in mice and gestation day 32 in humans, the ventral-lateral ectoderm surface of the frontonasal process (FNP) thickens and forms the nasal placodes (NPs). Around the NPs, the medial and lateral nasal processes (MNPs and LNPs) outgrow in a horseshoe shape, forming the nasal pits. At E10.5 in mice and gestation day 35 in humans, the MxPs show rapid lateral growth and push the nasal pits toward the LNPs; by E11.0 in mice and gestation day 38 in humans, the MxPs and the MNPs push the LNPs rostrally and fuse to form the upper lip. Epithelial seams between the MxPs, MNPs, and LNPs completely disappear by E11.5 in mice and gestation day 42 in humans. The MxPs further push the nostrils toward the median, and the entire process of upper lip formation is completed by E12.5 in mice and gestation day 48 in humans. Any failure in the fusion of these processes leads to a cleft in the upper face: a failure in fusion between the MxPs and the LNPs causes an oblique cleft and a failure in fusion between the MxP and the MNP causes a unilateral or bilateral cleft lip. Undergrowth of the FNP or fusion defects between the MNPs cause a midline cleft, whereas a failure in fusion between the MxPs and the mandibular process causes a transverse cleft. A cleft in the upper lip disconnects the orbicularis oris muscle, which plays important roles in closing the mouth, pursing the lips, and sucking. Therefore, surgical correction aims to improve both the aesthetic appearance and muscular dysfunctions.

In humans and mice, the palate (the roof of the oral cavity) is divided into two parts according to the anatomical origin. The primary palate (a.k.a. the anterior palate) is derived from the MNPs containing teethed incisors and canines, and the secondary palate originates from the MxPs containing premolars/molars. The anterior two-thirds of the palate constitute the hard palate, which is composed of bony elements, and the posterior one-third is called the soft palate and comprises five skeletal muscles (i.e., the *tensor veli palatini*, *levator veli palatini*, *palatoglossus*, *palatopharyngeus*, and *muscle uvulae*) that play crucial roles in swallowing, speech, and velopharyngeal closure. Surgical correction of both muscle disconnection and direction is important to restore proper muscular function.

Palatogenesis starts at E11.5 in mice and the sixth week of gestation in humans. The distal part of the MNPs develop into a pair of the intermaxillary segments and outgrow into the oral cavity to form the primary palate; on the other hand, lateral growth of MxPs results in a pair of palatal shelves by E12.5 in mice and the seventh week of gestation in humans. The palatal shelves grow vertically along with the sides of the tongue and then, following the downward tongue and jaw movement, elevate horizontally above the dorsal surface of the tongue. Cell proliferation and extracellular matrix (ECM) secretion/remodeling, which are regulated by growth factors and their signaling pathways, contribute to the growth of the palatal shelves during development. The growing palatal shelves meet at the midline of the oral cavity during E14.0–E14.5 in mice and the 7–9th week of gestation in humans. The medial edge epithelium (MEE) seam of the palatal shelves disappears through a combination of epithelial cell migration toward the nasal and oral epithelial triangles, apoptosis, and epithelial-to-mesenchymal transition (EMT) by E16.5 in mice and by the twelfth week of gestation in humans. Any failure in these steps causes a cleft in the secondary palate [6,7]. CPO can be categorized as complete, partial (location at either primary, secondary, or soft palate), or submucous. Submucous cleft palate does not display obvious clefts (a tissue gap) on the palate, but the palatal processes of the maxilla and palatine bones in the hard palate and/or the muscles in the soft palate are hypoplastic and/or disconnected at the palate midline due to persistence of MEE. Therefore, submucous cleft palate results in dysfunctions such as velopharyngeal incompetence and dysphemia.

Zebrafish (*Danio rerio*) are also widely used as an animal model in developmental research. Although the shape and components of craniofacial structures differ anatomically and morphologically from those in mammals, some of them show common functions and origins. For example, the ethmoid plate is a cartilaginous structure, which forms the roof of the oral cavity (like the hard palate) in mammals. The ethmoid palate and mammalian hard palate develop from cranial neural crest (CNC)-derived chondrocytes and mesenchymal cells, respectively. The molecular mechanisms and gene regulatory networks in craniofacial development, as well as histological and functional aspects, are conserved across species. Therefore, genetically modified zebrafish models are widely used to investigate developmental defects, including cleft lip and palate [8,9].

**Figure 1:**
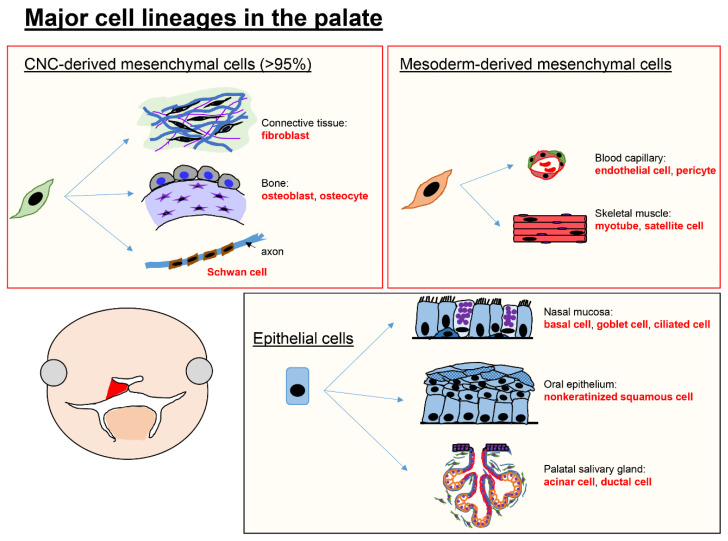
Major cell types in the palate. The majority of the mesenchyme of the lip and palate is composed of cranial neural crest (CNC) cells, which can form both bone and connective tissues. Epithelial cells develop into nasal and oral epithelial cells, characterized by different functions and gene expression profiles.

The lip and palate include several cell types derived from CNC cells, mesoderm-derived mesenchymal cells, and epithelial cells (Figure 1). In the palatal shelves, CNC cells give rise to fibroblasts in connective tissues, osteoblasts and osteocytes in bones, as well as Schwan cells, which wrap around axons and act as insulators for nerve transmission in the peripheral nervous system. Mesoderm-derived mesenchymal cells give rise to endothelial cells and pericytes in blood capillaries and myoblasts and satellite cells in skeletal muscles. Finally, epithelial cells give rise to basal cells, goblet cells, and ciliated mucous cells in the nasal mucosa, nonkeratinized squamous cells in oral epithelium, and acinar and duct cells in palatal salivary glands (the minor salivary glands located on the palate). Recent advanced technologies, including RNA sequencing at the single-cell level, allow us to identify not only novel cell populations and their fates in development but also cell-type-specific gene regulatory networks for cell specification and function.

As stated above, both genetic and environmental factors can contribute to CL/P cases in humans. Several potential non-genetic risk factors have been reported: cigarette smoking [10,11], alcohol consumption [12,13], obesity [14,15], high dietary glycemic index [16], and abnormal nutrient/vitamin conditions [17,18,19]. Moreover, appropriate folic acid supplementation can reduce the risk of developing spina bifida and CL/P in humans [20,21]. It is also known that some chemicals and drugs cause mutagenesis (i.e., they act as mutagens)**,** but some do not directly induce genetic mutations [22]. Therefore, there is the possibility that some substances may increase or decrease the risk for CL/P through epigenetic mechanisms such as regulation of non-coding RNAs, including microRNAs (miRNAs), transfer RNAs, ribosomal RNAs, small interfering RNAs, and long non-coding RNAs, as well as chromatin modifications such as methylation and acetylation.

miRNAs are single-strand non-coding RNAs containing 21–23 nucleotides that can anti-correlatedly and post-transcriptionally regulate the expression of multiple target genes [23,24,25]. miRNAs are transcribed as double-strand pri-miRNA and then cleaved by the DROSHA/DGCR8 complex to generate pre-miRNAs in the nuclei. pre-miRNAs are translocated to the cytoplasm by exportin-5 (XPO5) and cleaved by DICER, an enzyme crucial for miRNA maturation, to form miRNA/mRNA duplexes. Eventually these duplexes attach to Argonaute, a part of the RNA-induced silencing complex (RISC), resulting in loss of one strand and generation of mature miRNAs, which can bind to the 3′-untranslated region (UTR) of the target mRNAs [26,27]. miRNA biogenesis is conserved across species [28]. Importantly, there are multiple binding sites for different miRNAs on the 3′-UTR of the gene; therefore, gene expression is influenced by multiple miRNAs in a spatiotemporal manner. Accumulating evidence indicates that miRNAs play a crucial role in embryogenesis and that altered miRNA expression is associated with various birth defects [29]. In agreement with the importance of miRNAs and their processing enzymes in normal craniofacial development and CL/P in humans [30,31,32,33], mice with a deficiency for *Dicer* (*Dicer^F/F^;Wnt1-Cre* and *Dicer^F/F^;Pax2-Cre* conditional knockout mice) display severe craniofacial deformities, including cleft palate in both primary and secondary palates [34,35,36]. In zebrafish, mutants homozygous for point mutation *dicer1^sa9205^* exhibit smaller eyes, craniofacial dysmorphism, and aberrant pigmentation, thus resembling the mouse phenotypes [37].

**Figure 2:**
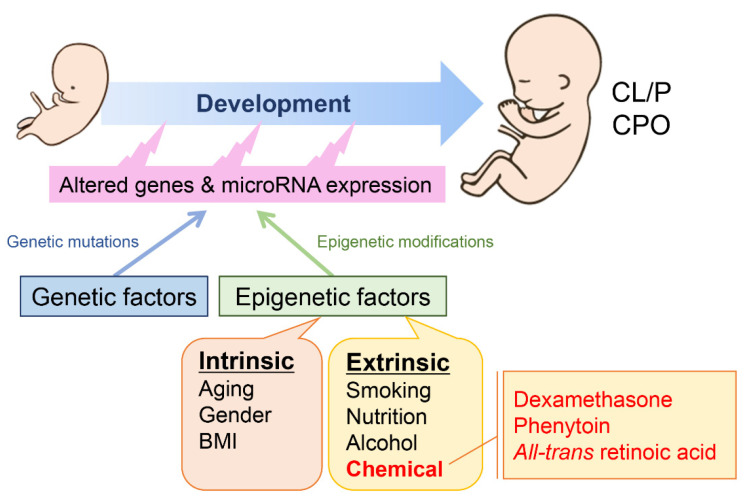
The cause of cleft lip with/without cleft palate (CL/P) and cleft palate only (CPO). Both genetic and environmental factors can contribute to the etiology of clefts. Environmental factors can alter the epigenetic status, including miRNA expression, DNA methylation, and chromatin modification. These epigenetic factors can be categorized into two groups: intrinsic and extrinsic factors. Chemical-induced cleft models are useful to study the contribution of pathogenic miRNAs to cleft lip and cleft palate.

In the past decade, an increasing number of studies have showed that expression of some miRNAs is drastically altered under pathological conditions [38,39]. These so-called pathogenic miRNAs may suppress genes that are crucial for development and homeostasis, affecting prognosis, drug resistance, and morphogenesis (Figure 2). Several studies have used RNA-seq to identify miRNA expression during normal lip/palate development as well as in non-syndromic CL/P [40,41]. In addition, mice with loss of function of miRNAs (*Dicer1^F/F^;Wnt1-Cre*) display severe craniofacial anomalies [35], indicating that some miRNAs are crucial for normal craniofacial development. An increasing number of studies with wild-type mice treated with specific inhibitors for each miRNA may provide some perspective on how an adequate expression of miRNAs is essential for normal orofacial development.

## 2. microRNAs Related to Cleft Lip

**Figure 3:**
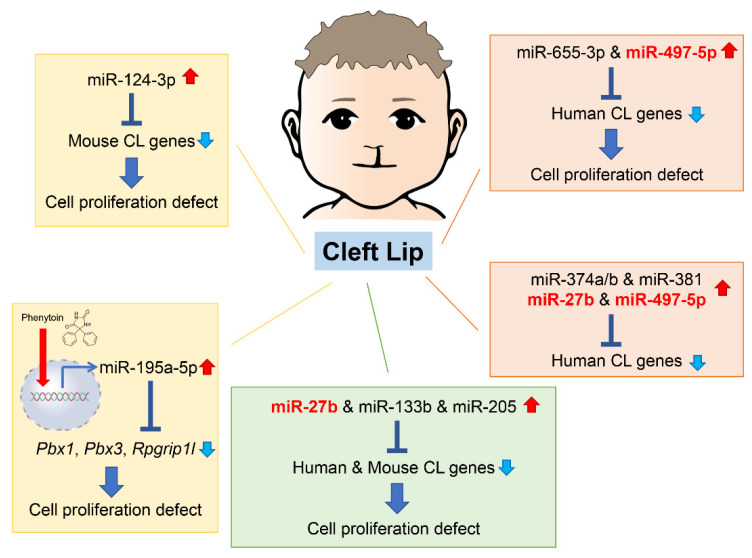
Summary of the miRNAs and genes associated with cleft lip in humans and mice. Phenytoin is a known inducer of cleft lip in mice. It inhibits cell proliferation in cultured cells through induction of pathogenic miR-196a-5p, which suppress expression of genes related to cleft lip. CL, cleft lip.

As of 2022, 55 mouse genes and more than 400 human genes had been reported as related to cleft lip and palate [42,43] in the gene datasets available at CleftGeneDB (https://bioinfo.uth.edu/CleftGeneDB/index.php?csrt=15984704412663399126, accessed on 28 October 2022). Bioinformatic analysis and consequent experimental validation identified miRNA-mediated gene regulatory networks in cleft lip (Figure 3). For instance, mmu-miR-124-3p suppresses cell proliferation in cultured mouse embryonic lip mesenchymal (MELM) cells through downregulation of cleft lip-related genes *Bmpr1a, Cdc42, Itf88, Pbx3*, and *Tgfbr1* [42]. In agreement with this function in MELM cells, mmu-miR-124-3p can suppress cell proliferation in other cell types, for instance, human keratinocytes (HaCaT) through *FGFR2* [44], human non-small cell lung cancer and nasopharyngeal carcinoma cells through *STAT3* [45,46], and colorectal cancer cells through *PRPS1* [47]. Under physiological conditions in C57BL/6J mice, mmu-miR-124-3p expression in the MxPs is upregulated at E12.5 and E13.5 compared to E10.5 and E11.5 [42]. This suggests that miR-124-3p is expressed at very low levels during normal lip development.

In our previous studies, we identified five miRNAs that regulate the expression of genes related to cleft lip. These miRNAs have not yet been reported or investigated in embryogenesis and craniofacial development. However, they are suggested to be associated with cancer pathogenesis and prognosis through changes in cell proliferation and differentiation. Since these miRNAs are specifically expressed under specific pathological conditions, such as cancer and cleft lip, they are considered to be pathogenic miRNAs related to cleft lip. Specifically, overexpression of hsa-miR-655-3p and hsa-miR-497-5p inhibits cell proliferation in cultured human lip mesenchymal cells through downregulation of cleft lip-related genes: *BCL1*, *CYPLA1*, *DMD*, *FZD6*, *HOXB3*, *MID1*, *NTN*, and *SATB2* by hsa-miR-655-3p; and *BAG4*, *CHD7*, *FGFR1*, *FOXP2*, *HECTD1*, *RUNX2*, and *TFAP2A* by hsa-miR-497-5p [43]. hsa-miR-665-3p decreases cell viability by apoptosis or suppresses cell proliferation through downregulation of target genes in various cells, namely *BCL2* in human lung adenocarcinoma cells [48], *NHEG1* in human neuroblastoma [49], *TRIM24* in human castration-resistant prostate cancer [50], and *FZD4* in human oral squamous cell carcinoma [51]. In addition, hsa-miR-497-5p inhibits cell proliferation through downregulation of target genes in several human cancer cells, e.g., *MAPK1* in cervical cancer cells [52], *PDL1* or *SLC7A5* in human colorectal cancer cells [53], and *WNT3A* in human nasopharyngeal carcinoma cells [54]. Thus, miR-124-3p, miR-655-3p, and miR-497-5p may play a key role in cell proliferation as tumor suppressors in cancers and CL/P inducers in development.

Interestingly, in the miRNA, transcription factor (TF), and non-TF networks, there is a common consensus subnetwork consisting of five TF genes (*GLI2*, *PAX3*, *PAX7*, *PAX9*, and *SATB2*), three non-TF genes (*FGFR1*, *RARA*, and *SUMO*), and five miRNAs (miR-27b, miR-133b, miR-205, miR-376b, and miR-376c) in humans and mice [55]. In cultured human and mouse lip mesenchymal cells, miR-27b inhibits cell proliferation through gene suppression of *PAX9* and *RARA*; miR-133b inhibits cell proliferation through gene suppression of *FGFR1*, *PAX7*, and *SUMO1*; and miR-205 inhibits cell proliferation through gene suppression of *PAX9* and *RARA* [55]. miR-27b-3p has been reported to be a tumor suppressor, inhibiting cell proliferation and migration through target gene expression in several cancer cells: *TAB3* in hepatocellular carcinoma [56], *MLL4* in glioblastoma stem cells [57], *TMED5* in gastric cancer cells [58], and *CTNNB1* in ovarian endometrial cells [59]. Overexpression of miR-133b suppresses cell proliferation viability and migration in various cancer cells: prostatic carcinoma cells through *ZNF587* [60] or *SDCCAG3* expression [61], cervical cancer cells through *ARFGEF1* expression [62], and lung adenocarcinoma through *CDCA8* expression [63]. Interestingly, miR-133b is upregulated in the exosomes secreted from skeletal muscle cells in limb and trunk muscles during development, regulating expression of the serum response factor (SRF) and myoblast differentiation in mice [64,65,66]; miR-133b is also thought to contribute to lip muscle development. miR-205 suppresses cell proliferation and migration in breast cancer cells through *KDM4A* [67], glioma cells though *VEGFA* [68], and gastric cancer cells through *FAM84B* [69], and miR-205-3p is downregulated in the nucleus pulposus of the intervertebral disc, which derives from the notochord, in mouse models for intervertebral disc degeneration [70]. Moreover, miR-205-3p suppresses WNT/β-catenin signaling, resulting in suppression of cell proliferation and ECM synthesis [70].

The miRNAs described above can commonly inhibit angiogenesis through downregulation of target genes. In fact, miR-205 downregulates *VEGA* in gastric cancer [71], hepatocellular carcinoma [72], and the extracellular vesicles from diabetic ulcers [73], whereas miR133b in the exosomes secreted from bone marrow mesenchymal stem cells downregulates *FBN1* [74] and miR-27b downregulates *AMPK* in brain microvascular endothelial cells [75], *CDH5* (a.k.a. VE-cadherin) in ovarian cancer [76], and *VEGFC* in gastric cancer [77]. Since angiogenesis is critical for tissue growth and development, these miRNAs may play a role in various tissue processes from morphogenesis through angiogenesis.

## 3. microRNAs Related to Cleft Palate

**Figure 4:**
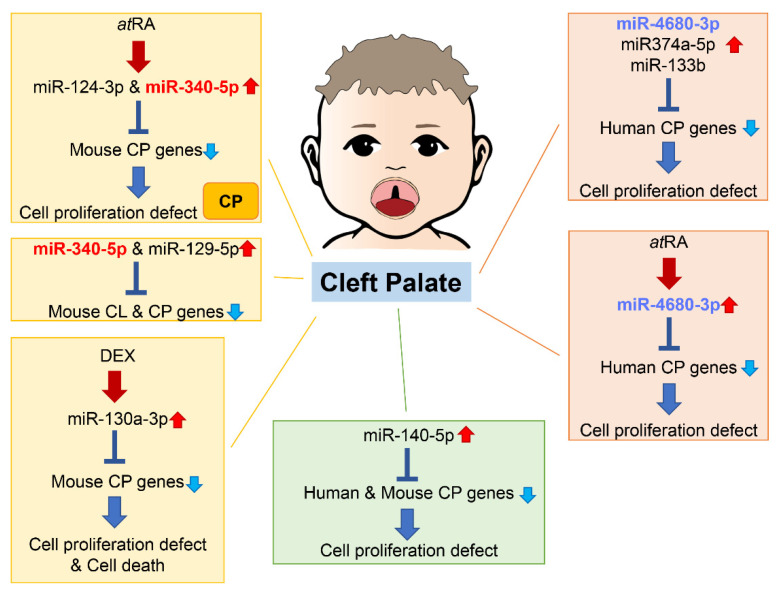
Summary of miRNAs and genes related to cleft palate in humans and mice. Through bioinformatic analysis for gene expression datasets and genes related to cleft palate, several miRNAs related to cleft palate are suggested to be pathogenic miRNAs. Many miRNAs among them have been validated in cultured cells and/or chemical-induced cleft palate mouse models. CL, cleft lip; CP cleft palate (including CPO and CLP).

An increasing number of studies show that miRNAs are involved in both normal palate and CL/P development in humans and mice (Figure 4).

As of 2021, 395 genes (CPO: 367 genes; anterior cleft: 16 genes; posterior/soft palate cleft: 15 genes; submucous cleft: 37 genes; and CLP: 44 genes) were reported as genes related to cleft palate in mice and 131 genes in humans [78,79] (the updated list of genes is available at CleftGeneDB; Table 1). A total of 365 mouse strains show complete cleft of the secondary palate, 44 mouse strains exhibit CLP, 14 mouse strains display anterior cleft palate, 16 mouse strains present posterior cleft palate (soft palate cleft), and 37 strains have submucous cleft palate. Overexpression of miR-374a-5p, miR-4680-3p, and miR-133b suppresses cell proliferation through the regulation of genes related to human cleft palate in cultured human palatal mesenchymal cells: *ARNT*, *BMP2*, *CRISPLD1*, *FGFR2*, *JARID2*, *MSX1*, *NOG*, *RHPN2*, *RUNX2*, *WNT5A*, and *ZNF236* by miR-374a-5p; *ERBB2*, *JADE1*, *MTHFD1*, and *WNT5A* by miR-4680-3p; and *FGFR1*, *GCH1*, *PAX7*, *SMC2* and *SUMO1* by miR-133b [78].

Overexpression of miR-374-5p suppresses cell proliferation in several cells: in human non-small cell lung carcinoma cells by suppressing *NCK1* expression [80], and in human neural stem cells by suppressing *HES1* expression, which promotes neural stem cell differentiation [81]. On the other hand, miR-374-5p shows protective effects in cell viability, reducing apoptotic cell death induced by either oxygen/glucose deprivation (an infant hypoxic-ischemic encephalopathy model) in rat PC12 neuronal cells [82] or by LPS in human pulmonary microvascular endothelial cells [83]. Interestingly, maternal circulating hsa-miR-374-5p is strongly associated with the risk of small-for-gestational-age birth and preterm delivery in humans [84,85], suggesting that miR-374-5p may influence cell proliferation and survival in development.

A total of 44 cleft palate genes are common in humans and mice. A bioinformatic analysis revealed that miR-140-5p is a potential pathogenic miRNA that specifically induces cleft palate in both humans and mice [86]. Overexpression of miR-140-5p suppresses genes that are crucial for palate formation (*Pdgfra* for the primary palate, *Pax9* for the secondary palate, and *Bmp2* and *Fgf9* for both primary and secondary palate) in human and mouse palatal mesenchymal cells. However, the role of miR-140-5p seems to vary per cell type. Its overexpression induces adipogenic differentiation and lipogenesis through suppression of PDGFRα in pre-adipocytes [87] and alleviates pyroptosis by targeting *Ctsb* in chondrocytes treated with LPS (an osteoarthritis (OA) model) and in articular cartilage in OA mice [88]. On the other hand, overexpression of miR-140-5p suppresses osteogenic differentiation by targeting *SATB2*-mediated ERK1/2 and P38MAPK signaling pathways in human vascular smooth muscle cells [89]. Moreover, miR-140-5p binds to NRF2, which is a key molecule for anti-oxidative stress and cellular toxicity, enhances the NRF2/HO-1 signaling pathway, and suppresses cell proliferation, cell migration, and angiogenesis in breast cancer cells under hypoxia conditions [90]. In zebrafish, overexpression of miR-140 results in a cleft between lateral elements of the ethmoid plate, a structural analog of the palate in higher vertebrates, through the suppression of *Pdgfra* [91]; in mice, *miR-140* null mice exhibit submucous cleft palate with hypoplastic palatal bones [92]. Thus, a fine-tuned, precise amount of miR-140 would be crucial for palate development. A single nucleotide polymorphism (SNP) in pre-miR-140 responsible for decreasing miR-140-5p expression is associated with an increased risk of non-syndromic CL/P (nsCL/P) in humans [93]. SNPs in *PDGFRA* are also associated with risk of developing nsCL/P, with one SNP found at the 3′-UTR near a binding site for miR-140 [94]. These results suggest that the miR-140–PDGFRA axis plays a crucial role in CL/P.

Mutations in *TBX1* cause CL/P or CPO in humans and mice [95,96,97], whereas overexpression of *Tbx1* suppresses *Zeb2* expression in Hela cells, which induces EMT and reduces stemness [98,99], cell proliferation, and keratinocyte differentiation [100]. TBX1 binds to the 3′-UTR of a miR200b/200a/429 cluster (an EMT suppressor) and induces miR-200b/200a/429 expression, resulting in the suppression of *Zeb2* and miR-203 in Hela and A549 cells [98]. miR-200b/200a/429/miR-203 negatively regulates *Zeb2* expression. As expected, expression of miR-200b-5p, 429-3p, and 203-3p is significantly downregulated in palatal epithelial cells, and expression of *Zeb1* and *Zeb2* is upregulated in the developing palate in *Tbx1* null mice. These findings suggest that the TBX1–miR-200b/200a/429 and miR-203–ZEB2 loop is important for epithelial cell differentiation, EMT, and stemness in the palatal epithelium, and their dysregulation results in CL/P. Indeed, *miR-17-92* null mice (*miR-17-92^-/-^*, *miR-17-9^-/-^;miR-106b-25^+/-^*, and *miR-17-92^-/-^;miR-106^-/-^* mice) display CLP through upregulation of *Tbx1*, *Tbx3, Fgf10, Shox2,* and *Osr1* expression [101]. On the other hand, overexpression of the miR-17-92 cluster suppresses expression of *E2F1*, a transcription factor, and inhibits cell proliferation through dysregulation of the cell cycle in mouse embryonic palatal mesenchymal (MEPM) cells [102]. Moreover, transgenic mice expressing inhibitors for miR-17-92 and miR-17-18 exhibit complete CPO through upregulation of *Tgfbr1* and *Tgfbr2* expression [103].

Human linkage analyses suggest that mutations in non-coding miRNA regions are associated with susceptibility to nsCL/P. For instance, miR-152 hypomethylation leading to overexpression is frequently detected in nsCL/P, and overexpression of miR-152 in zebrafish results in craniofacial cartilage dysmorphism [104]. An SNP in rs539075, located in the *CDH2* intron where it is suggested to encode miRNAs, is associated with nsCL/P [105]. Mutations in *CDH2*, which plays a role in EMT, cause syndromic or non-syndromic Peters anomaly, characterized by corneal opacity, hypertelorism, and thin upper lip [106]. Thus, some SNPs are related to the production of miRNAs, while others are related to the binding of miRNAs. For instance, several intronic SNPs located within or near miRNA-binding sites (rs1048201/miR-496 in *FGF2*, rs3733336/miR-145 in *FGF5*, and rs546782/miR-187 in *FGF9*) are suggested to constitute a risk for nsCL/P [107]. rs12532 within the 3′-UTR of *MSX1* may affect the binding to miR-3649, leading to a decrease in risk of developing nsCL/P through the regulation of *MSX1* expression [108]. Interestingly, miR-let7-3p expression is downregulated in both the plasma from mothers carrying a nsCL/P fetus and lip tissues from nsCL/P individuals [109]. The inhibition of miR-let7-3p suppresses cell proliferation through *HHIP* upregulation and *GLI2* downregulation in human oral keratinocytes. Thus, maternal miR-let-3p expression may become a potential diagnostic biomarker for nsCL/P during pregnancy. Interestingly, expression of miR-378 shows sex differences (i.e., downregulated in female nsCL/P individuals and upregulated in males) [110]. Increasing evidence suggests that maternal miRNA expression and SNPs in miRNA biogenesis enzymes or the 3′-UTR of CL/P-associated genes can be used for screening CL/P during pregnancy. To date, each miRNA-specific inhibitor or mimic, which can modify miRNA expression independently, is developed industrially. Several researchers have succeeded in inducing or rescuing developmental defects by administering these inhibitors/mimics to pregnant mice or zebrafishes. In the near future, these techniques can be applied to repair or reduce the severity of CL/P during pregnancy in humans.

## 4. microRNAs Involved in Chemical-Induced Cleft Lip and Cleft Palate

The underlying pathogenic mechanisms in CL/P and CPO are complicated by both genetic and non-genetic factors. Human cohort studies show that maternal exposure to several drugs and chemicals that act as teratogens induces nsCL/P [111,112]. For example, dioxins/TCDD (2,3,7,8-tetrachlorodibenzo-*p*-dioxin) [113], phenytoin [114], antibiotics [115], corticosteroids [116], smoking [117], a high dose of alcohol [12,118], and heavy metals [119] are known teratogens for nsCL/P. Human linkage analyses show that mutations in genes related to TCDD metabolism (*AHRR*, *ARNT*, and *CYP1A1*) and a copy number change in *AHR* are associated with increased risk of CL/P [120,121]. Moreover, mutations in *CYP1A1* and *GSTT1* in combination with maternal smoking increase the risk of developing CL/P in humans [122,123]. These findings suggest that gene–environment interactions contribute to the pathogenesis, susceptibility, and prevention of CL/P.

Non-coding RNAs and methylation status may explain how CL/P-associated gene expression is altered by teratogens. Exposure to several chemicals (e.g., retinoic acid, dexamethasone, dioxins) induces cleft palate in mice and in humans [124,125,126]. Retinoic acid (*at*RA) induces expression of miR-124-3p [127,128] and miR-106-5p [129] in cultured MEPM cells and the developing palatal shelves in mice. miR-124-3p can inhibit cell proliferation through suppression of genes crucial for palate development, and miR-106-5p induces apoptosis and compromises phosphatidylcholine synthesis/cell membrane synthesis though suppression of *Tgfbr2*. Importantly, a specific inhibitor for miR-124-3p normalizes cell proliferation under *at*RA treatments and prevents cleft palate in 65% of *at*RA-induced cleft palate mice. More recently, another candidate miRNA, miR-340-5p, was identified in *at*RA-induced cleft palate mice [128]. Therefore, treatment with a combination of miR-124-3p and miR-340-5p inhibitors can prevent cleft palate with almost full penetrance [128]. This suggests that it is possible to prevent CL/P by normalizing maternal pathogenic miRNA expression. Dexamethasone, on the other hand, inhibits cell proliferation through miR-130-3p induction, which suppresses *Slc24a2* expression, in cultured MEPM cells [130]. Overexpression or downregulation of miR-130-3p induces or suppresses cell proliferation, migration and invasion, respectively [131,132], whereas its suppression inhibits cell proliferation, TNFα-induced cell migration, and pro-inflammatory cytokine production in MH7A cells (a human rheumatoid arthritis synovial cell line) though upregulation of *KLF9* [133].

In mice, exposure to phenytoin is related to cleft lip [134]. Phenytoin induces miR-196a-5p expression and inhibits cell proliferation through the suppression of *Pbx1*, *Pbx3*, and *Rpgrip1l* in cultured MELM cells [135]. In the MxPs and the NPs, miR-196a-5p expression drastically drops down during E10.5 to E12.5 [135]. miR-196a-5p suppresses cell proliferation and promotes osteogenic differentiation in human Wharton’s jelly umbilical cord stem cells (WJCMSC) and suppresses bone formation in WJCMSC-sheet transplanted rat calvaria through suppression of *Serpinb2* [136]. Moreover, it causes an imbalance in proliferation and apoptosis through *Foxo1* expression in vascular smooth muscle cells treated with oxidized low-density lipoprotein [137], and inhibits cell proliferation, migration, and tumor invasion in several cancer cells [138,139,140]. Co-transfection of miR-196a-5p/10b-5p/615-3p induces the fate determination of paraxial mesodermal cells and skeletal muscle differentiation in embryonic stem cells [141]. miR-196a-5p in extracellular vesicles secreted from myoblasts inhibits osteoclastogenesis through a reduction in mitochondrial energy metabolism in mouse pre-osteoclastic Raw264.7 cells, while it promotes osteoblastogenesis in MC3T3-E1 cells [142]. miR-196a-5p also induces osteogenic and adipogenic differentiation in mesenchymal stem cells derived from the bone marrow [143]. Taken together, miR-196a-5p may be involved in various developmental processes during palate formation.

In summary, modulation of miRNA expression may be key in understanding the toxicity of chemicals and congenital birth defects. In this review, we discussed selected CL/P mouse models and speculated that expression of some miRNAs is commonly altered by exposure to various chemicals. If we can detect these unique pathogenic miRNAs before or during pregnancy, they may become new biomarkers for diagnosis and potential therapeutic targets to prevent or reduce the risk of chemical-related birth defects.

## 5. Conclusions

An increasing number of studies suggest a contribution of miRNAs to cleft lip and cleft palate development in humans and mice. Bioinformatic approaches using both sequencing (miRNA-seq and mRNA-seq) and reported cleft-related genes are striking in the identification of miRNAs related to cleft palate. In addition, chemical-induced cleft models can help us identify the underlying mechanisms and allow us to test potential clinical interventions to prevent cleft lip and cleft palate.

## Figures and Tables

**Table 1:** Genes related to orofacial cleft.

Cleft Type (# of Genes)	Mouse Type	Genes
Complete CPO (367 genes)	Single gene mutation	*Acan Acvr1 Acvr2a Adamts3 Adamts6 Adamts20 Adgra2 Afdn Amer1 Anp32b Ap2b1 Arhgap29 Asxl1 Barx1 Bcor Bmp2 Bmp4 Bmp7 Bmpr1a Bnc2 Cask Casp3 Ccn2 Ccp110 Cdc42 Cdk20 Cdkn1c Chd7 Chrd Chuk Col2a1 Colgalt1 Crampl Crebbp Crk Ctnnb1 Ctnnbip1 Cycsp Cyp26b1 Cyp51 Dhcr7 Dhrs3 Dicer1 Dlg1 Dlx1 Dlx2 Dlx5Dnmt3b Dph1 Edn1 Edn2 Ednra Efnb1 Efnb2 Egfr Ermp1 Esrp1 Eya1 Fam20b Fbxo11 Fbxw7 Fgf8 Fgf9 Fgf10 Fgf18 Fgfr1 Fgfr2 Fgfr2c Fign Flna Foxc2 Foxe1 Foxf2 Fras1 Fst Fuz Fzd2 Gab1 Gabrb3 Gad1 Gas1 Gbx2 Gdf11 Glce Glg1 Gli2 Golb1 Gpc6 Grb2 Grhl3 Gsk3b Gskip Haao Hand2 Has2 Hdac3 Hoxa2 Hoxb7 Hs2st1 Hsd17b7 Hspb11 Hspg2 Ift88 Ift140 Igf2 Ilk Impad1 Inhba Inpp5e Irf6 Itga5 Itgav Ift140 Ift 172 Igf2 Ilk Impad1 Inha Inpp5e Irf6 Itga5 Itgb1 Itgb8 Jag2 Jmjd6 Kat6a Kcnj2 Kcnj13 Kdf1 Kif7 Kif20b Kifbp Ldb1 Lhx8 Loxl3 Lrp2 Luzp1 Map3k7 Mapk1 Med23 Megf8 Meis2 Men1 Meox2 Mfcs4 Midn Mirc1 Mir17-18 Msk1 Mn1 Mnt Msx1 Msx2 Mybphl Nabp2 Mectin1 Mectin4 Nog Nosip Nprl3 Nrp1 Nsd2 Nxn Oca2 Ofd1 Osr2 Pax3 Pax9 Pcnt Pcsk5 Pdgfc Pdgfra Pds5a Pds5b Pdss2 Phc1 Piga Pigv Pitx1 Pitx2 Pkdcc Plod3 Plxnd1 Pnn Porcn Prdm16 Prickle1 Prrx1 Ptch1 Pygo2 Qrich1 Qsox1 Rad23b Rbfox2 Rdh10 Recql4 Robo1 Ror2 Rpgrip1l Rspo2 Runx2 Ryk Ryr1 Satb2 Sc5d Sclt1 Serpinh1 Sfn Sh3pxd2a Shh Sim2 Skor2 Slc13a4 Slc32a1 Slc35d1 Slmap Smad7 Smo Smoc1 Snai2 Snx3 Sos1 Sox2 Sox5 Sox9 Sox11 Spry2 Sufu Sumo1 Tapt1 Tbc1d32 Tbx1 Tbx2 Tbx22 Tcof1 Tctn2 Tent5c Tfap2a Tgds Tgfb2 Tgfb3 Tgfbr2 Tgfbr3 Tmem107 Trppc10 Trp53 Trp63 Trps1 Ttc21b Twist1 Ugdh Vax1 Vegfa Wdpcp Wdr19 Wls Wnt5a Wen Zeb1 Zmynd11*
	Spontaneous	*Abn Acan Am Cacnal2 Col11a1 Crn Csp2 Far Fgf9 Gli3 Hpmd Lmbr1 M9bei Mut1679 Oca2 Oel Pad Pc Pcp* *Ptd Rpl38 Sho Sme Srn Srt Ur Zeb1*
	Compound mutant	*Adamts9;Adamts20 Adamts20;Ptch1 Adamts20;Vcan Akap8;Fign Arid5b;Pdgfra Ard5b;Zfp950 Bmi1;Pcgf2 Bmp2;Bmp4 Bmp4;Bmp7 Bmp2;Bmp4;Bmp7 Boc;Cdon Chrd;Nog Chrd;Tbx1 Dlx1;Clx2 Clx5;Msx1 Dph1;Ovca2 Dph1;Ovca2;Trp53 Ednrb;Spry2 Ephb2;Ephb3 Eya1;Six1 Eya1;Sumo1 Fgfr1;Fgfr2 Fuddle;TCZ-tau Fzd1;Fzd2 Fzd2;Fzd7 Fzd2;Vangl2 Fzd2;Fzd7;Wnt3a Fzd2;Fzd7;Wnt11 Gab1;Met Gad1;Gad2 Gas1;Shh Gdf11;Mstn Gdf11;Wfikkn1 Gdf11;Wfikkn2 Golga5;Golgb1 Gsc;Gsk3a H19;Igf2r Hspa5;TCZ-tau Hoxa1;Hoxa2 Igf2;Rr27 Inhba;Inhbb Insig1;Insig2 Irf6;Sfn Itga5;Itgav Itgb6;Itgb8 Kat6a;Tbx1 Kdf1;Sfn Kif20b;TCZ-tau Lbr;Tm7sf2 Lgr4;Lgr5;Lgr6 Lgr5;Lgr6 Lhx6;Lhx8 Lrp6;Rspo2 Mapk1;Mapk3 Mdm2;Mdm4 Mmp14;Mmp16 Msc;Tcf21 Ncor2;Ncor2 Nectin1;Nectin4 Osr2;Pax9 Pax9;Msx1 Pax9;Sostdc1 Pbx1;Pbx2 Pbx1;Pbx2;Pbx3 Pdgfra;Pdgfrb Pdgfra;Plekha1 Phc1;Phc2 Prrx1;Prrx2 Ptprf;Ptprs Pygo1;Pygo2 Ror1Ror2 Ror2;Wnt5a Ror1;Wnt9a Shh;Six3 Six1;Six4 Snai1;Snai2 Sox5;Sox6 Spry1;Spry2 Tbx2;Tbx3 Tfap2a;Tfap2b Tgfb1;Tgfb3 Vax1;Vax2 Yap;Taz*
Partial CPO: anterior (16 genes)	Single gene mutation	*Codn Ctnnb1 Fgfr2 Gsc Lims1 Runx1 Shh Shox2 Sox11 Tbx1 Tbx3 Tgfb3*
	Compound mutant	*Boc;Cdon Map3k7;Smad4*
Partial CPO: posterior/soft palate (15 genes)	Single gene mutation	*Bnc2 Dlx5 Foxf2 Hic1 Hox3a Mef2c Mfcs4 Pax3 Rspo2 Sim2 Smo Tbx1 Tgfbr1 Tgfbr2 Tshz1*
	Compound mutant	*Dlx5;Mef2c*
Submucous cleft palate (37 genes)	Single gene mutation	*Acvr1 Amer1 Apaf1 Arid5b Asph Bmp4 Csrnp1 Dlx5 Eda Eya4 Fras1 Inhba Krt5 Lrp4 Meis2 Ndst1 Nog Recql4 Schip1 Six3 Sgpl1 Smad4 Smo Sostdc1 Tbx1 Tbx3 Tbx22 Tgfb3 Tgfbr1 Tgfbr2 Tiparp Zfp640 Zfp950*
	Compound mutant	*Map3k7;Smad4 Shh;Six3 Smad4;Irf6 Smad4;Trim33*
CLO (23 genes)	Single gene mutation	*Bmp4 Cplane2 Ermp1 Folr1 Gli3 Kynu Mks1 Pbx1 Pgap2* *Ptch1 Rpgrip1l Sp8 Tbx1 Tgfbr1*
	Spontaneous	*Clf2 Knyn Rpl38 Wnt9b*
	Compound mutant	*Aldh1a2;Aldh1a3 Bbs7;Ift88 Gdf1;Nodal*
CLP (44 genes)	Single gene mutation	*Bmpr1a Cdc42 Cplane1 Ctnnb1 Dzip1l Ednrb* *Ermp1 Esrp1 Folr1 Ift88 Ihh Kif3a Kynu Lrp6 Mirc1 Mks1* *Pbx1 Pgap2 Rpgrip1l Rspo2 Sox11 Tfap2a Tgfbr1 Trp53* *Trp63 Ttc21b Wdr19 Wnt9b*
	Spontaneous	*Clf2 Knyn Rpl38 Tbx10 Zeb1*
	Compound mutant	*Bbs7;Ift88 Esrp1;Esrp2 Fgf8;Tfap2 Hhat;Ptch1 Lrp6;Rspo2 Mirc1;Mirc3 Msx1;Pax9 Pbx1;Pbx2 Pbx1;Pbx3 Pbx1;Wnt9b Rspo2;Wnt9b*

## Data Availability

Not applicable.
